# Some Recent Additions to the Materia Medica

**Published:** 1890-03

**Authors:** 


### SOME RECENT ADDITIONS TO THE MATERIA MEDICA.

Among the new drugs which Messrs. Parke, Davis & Co., announce they can supply, are the following:

### COCILLANA.

PROPERTIES.—Expectorant, tonic and laxative.

Dose, 10 to 30 minims.

### ESCHSCHOLTZIA.

PROPERTIES.—"An excellent soporific and analgesic, and above all, harmless"—may with advantage replace opium preparations for children. Fluid extract of the plant.

Dose, 15 to 30 minims.

### JATROPHA.

PROPERTIES..—Alterative and cholagogue; in larger dose hydro-cathartic and sometimes emetic. Fluid extract of the root.

Dose, 1-2 to 2 fluid drachms.

### ECHINACEA.

PROPERTIES.—Very strong claims have been recently made for this drug as an alterative of great value in all strumous and syphilitic indications. Old chronic wounds, such as fever sores, old ulcers, etc., have yielded to its use after resisting potassium iodide, sarsaparilla, yellow dock, etc.

It is also stated to be an excellent remedy in the treatment of blood-poisoning, of snake bites, and as a prophylactic and also curative agent in hydrophobia. Fluid extract of the root.

Dose, 1-4 to 1-2 fluid drachms.

### HYDRASTININE.

A new derivative of hydrastine. A possible substitute for ergot.

Recent advices from the highest European authorities represent it to be of immeasurable service in controlling uterine hemorrhages, far surpassing ergot in efficiency, certainty of action and safety.

BROOM-CORN SEED.

PROPERTIES.—Diuretic, sedative, demulgent and soothing to the irritated urinary organs in vesical catarrh, cystitis and irritable bladder.

In the aged who are compelled to rise frequently at night to void their urine, it has produced great relief. It must not be confounded with broom top or scoparius.

Dose of fluid extract of the seeds, 1 fluid drachm three to five times daily.

